# Evaluation of the effect of two biomimetic approaches on micro-tensile bond strength of one etch & rinse adhesive to dentin (An *in vitro* study)

**DOI:** 10.3389/fdmed.2026.1791403

**Published:** 2026-04-08

**Authors:** Ahmad Diaa El Din Abou El Seoud, Essam Abdel Hafez Naguib, Eman Ali Abou Auf, Sara Hany Younis

**Affiliations:** 1 Conservative Dentistry Department, Faculty of Oral and Dental Medicine, Future University in Egypt, Cairo, Egypt; 2 Department of Conservative Dentistry, Faculty of Oral and Dental Medicine, Future University in Egypt, Cairo, Egypt and Conservative Dentistry Department, Faculty of Dentistry, Cairo University, Cairo, Egypt; 3 Conservative Dentistry Department, Faculty of Dentistry, Cairo University, Cairo, Egypt

**Keywords:** adhesive dentistry, biomimetic remineralization, dentin bonding, micro-tensile bond strength, polyaspartic acid, polymer-induced liquid precursor, self-assembling peptide p11-4

## Abstract

**Background:**

Hydrolytic and enzymatic degradation of the resin–dentin interface compromises the longevity of adhesive restorations. Biomimetic strategies for guided dentin remineralization, including self-assembling peptide P11-4 (Curodont Repair™) and the polymer-induced liquid precursor (PILP) process using poly-L-aspartic acid, have been proposed to stabilize the collagen matrix and enhance adhesive bond durability.

**Methods:**

Fifty-four extracted human molars were artificially demineralized and randomly assigned to three pretreatment groups (*n* = 18 each): control (no pretreatment), self-assembling peptide P11-4 (Curodont Repair™, Credentis AG) applied for 5 min, or polymer-induced liquid precursor (PILP) solution containing 100 μg/mL poly-L-aspartic acid applied for 1 h. Each group was subdivided according to storage time in artificial saliva (24 h, 3 days, or 14 days) (*n* = 6 teeth per subgroup). Specimens were restored using an etch-and-rinse adhesive system (Adper Single Bond 2, 3M ESPE) and resin composite. One micro-tensile beam (≈1 × 1 mm^2^) was prepared per tooth (experimental unit = tooth). Micro-tensile bond strength (µTBS) was measured using a universal testing machine equipped with a 500 N load cell at a cross-head speed of 0.5 mm/min. Data were analyzed using two-way ANOVA and Bonferroni *post hoc* tests (*α* = 0.05).

**Results:**

Two-way analysis of variance revealed significant effects of pretreatment, storage time, and their interaction on µTBS values (*p* < 0.001). At T0 and T1, specimens pretreated with P11-4 exhibited the highest mean µTBS values, which were significantly greater than those of the PILP group and comparable to or higher than the control group. At T2, the control group demonstrated significantly higher µTBS values, while both biomimetic pretreatment groups showed a significant reduction in bond strength. Failure mode analysis revealed predominantly mixed failures at T0 and T1 across all groups, whereas adhesive failures were more prevalent at T2 in the biomimetic groups; the control group exhibited mixed and cohesive-in-dentin failures.

**Conclusions:**

pretreatment with the self-assembling peptide P11-4 resulted in higher micro-tensile bond strength to demineralized dentin compared with the PILP approach at early storage periods. However, the durability of both biomimetic dentin remineralization strategies was time-dependent**.**

## 1 Introduction

The long-term success of adhesive restorations is hindered by degradation of the hybrid layer formed between resin and dentin. This degradation is primarily caused by incomplete resin infiltration, hydrolytic stress, and enzymatic breakdown by endogenous dentin proteases such as matrix metalloproteinases (MMPs) and cysteine cathepsins (1, 2). These mechanisms compromise the bond integrity and lead to restoration failure (3).

**Figure 1:**
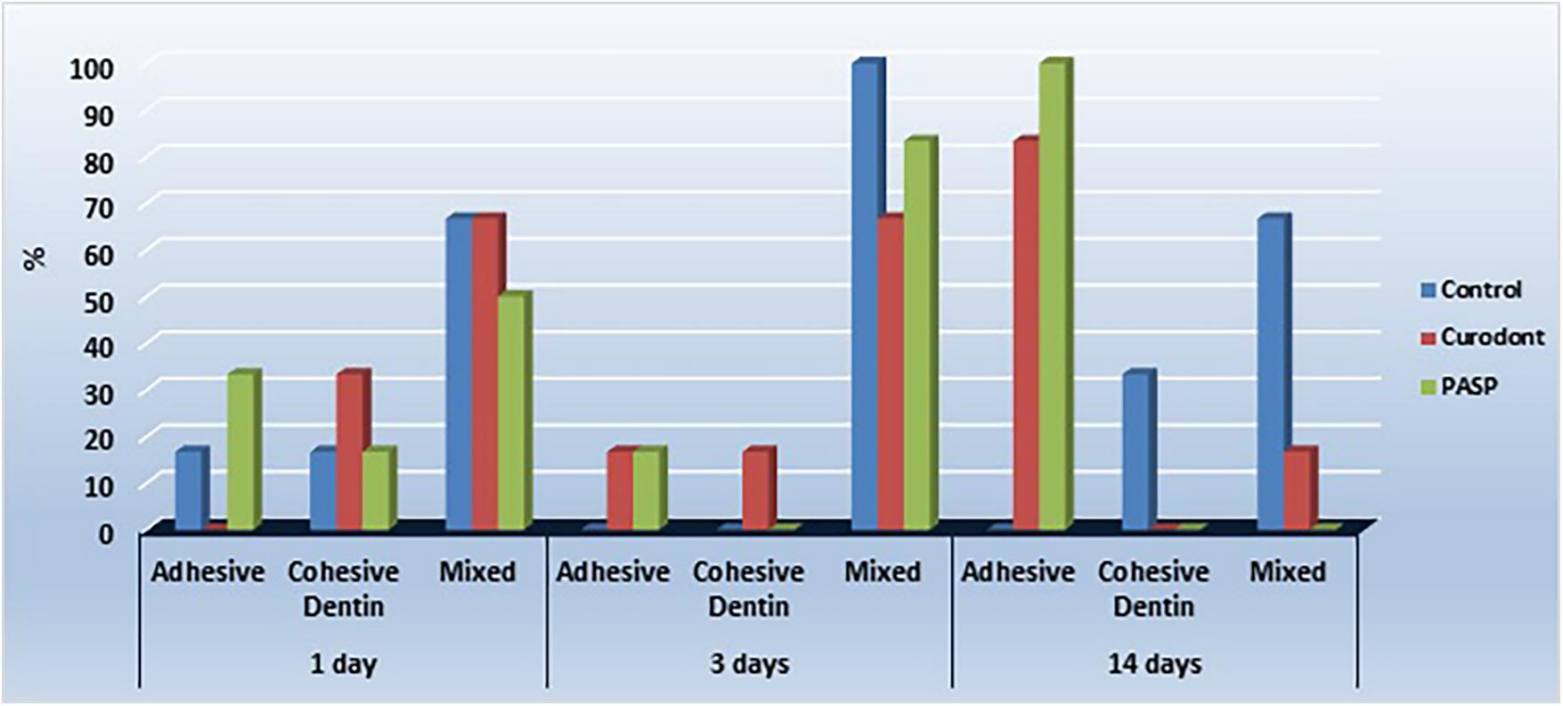
Micro-tensile bond strength (µTBS) values (MPa) of control, P11-4 (Curodont), and PILP (PASP) groups at different storage times (1 day, 3 days, and 14 days). Data are presented as mean ± standard deviation.

**Figure 2:**
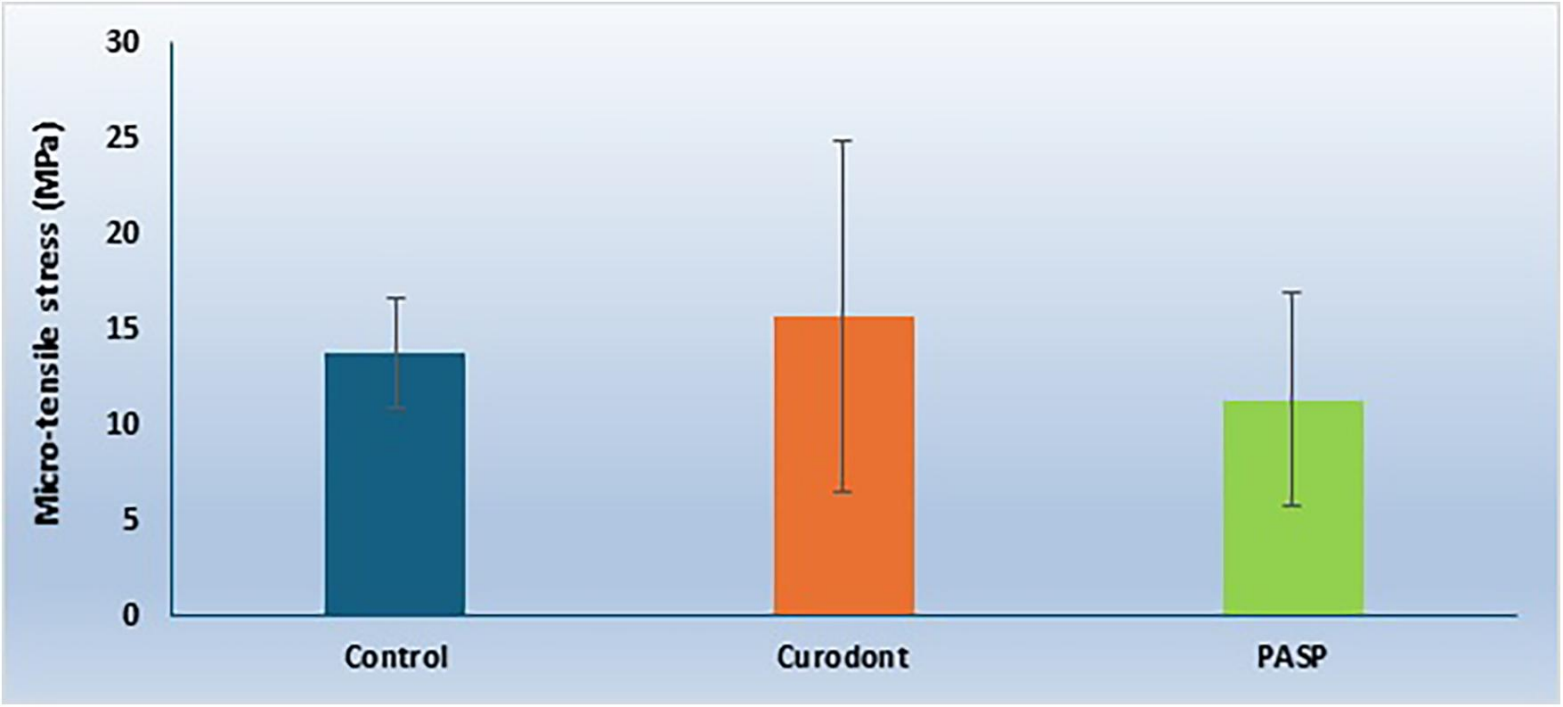
Distribution of failure modes (%) for control, P11-4 (Curodont), and PILP (PASP) groups at different storage times. Failure modes include adhesive, cohesive in dentin, and mixed failures.

**Figure 3:**
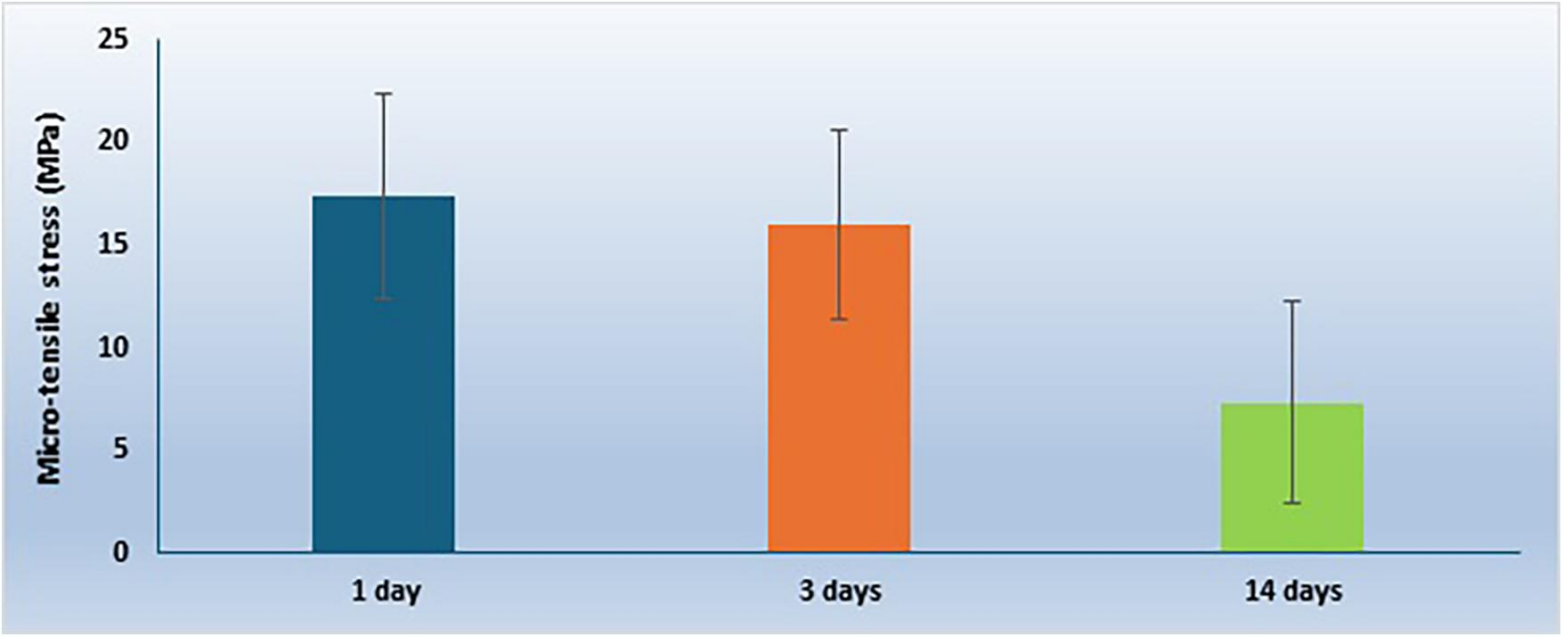
Overall comparison of mean micro-tensile bond strength (µTBS) values (MPa) among the three dentin pretreatment groups (control, P11-4, and PILP), irrespective of storage time. Data are presented as mean ± standard deviation.

**Figure 4:**
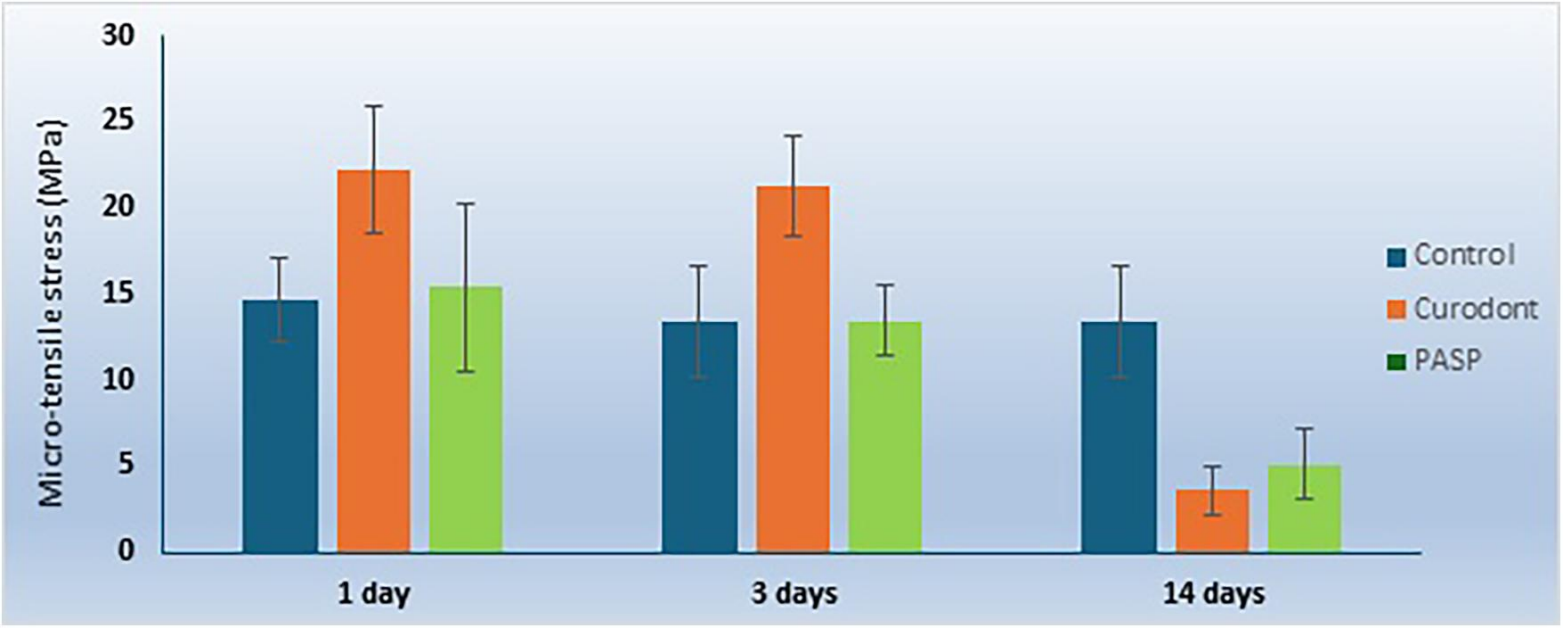
Effect of storage time on micro-tensile bond strength (µTBS) values (MPa), irrespective of dentin pretreatment group. Data are presented as mean ± standard deviation.

Biomimetic remineralization provides an alternative approach that aims to restore mechanical strength by promoting mineral deposition within the collagen matrix, mimicking natural biomineralization (4). Two such approaches are the self-assembling peptide P11-4 (Curodont Repair™), which serves as a nucleation scaffold for calcium phosphate crystallization (5), and the polymer-induced liquid precursor (PILP) process, which uses poly-L-aspartic acid to stabilize amorphous calcium phosphate nanoparticles, enabling infiltration into collagen fibrils (6).

Self-assembling peptide (P11-4) biomimics natural mineralization of collagen fibrils by attracting calcium ions, which prompts *de novo* crystallization of hydroxyapatite crystals. This occurs as P11-4 assembles in a pre-arranged hierarchical pattern producing three-dimensional fibrillar scaffolds that encourage hydroxyapatite crystallization. Although P11-4 has demonstrated the ability to control hydroxyapatite crystal growth and deposition on enamel, its behavior on dentin substrates and its interaction with adhesive systems remain less clearly understood (7).

In the extracellular matrix, protein phosphorylation is necessary for bone and dentin development, permitting mineralization of collagen fibers in both extrafibrillar and intrafibrillar spaces. The Polymer-Induced Liquid Precursor (PILP) approach attempts to imitate this process for collagen remineralization. This method depends on process-directing agents that stabilize saturated calcium phosphate solutions by forming calcium- and phosphate-rich nanodroplets. When exposed to collagen fibrils, these nanodroplets promote the formation of amorphous calcium phosphate that gradually transforms into apatite crystals, resembling native dentin (8). However, this approach has not yet been incorporated into commercially available products for dentinal caries management.

An etch-and-rinse adhesive system was selected in the present study to provide a standardized and extensively documented bonding protocol. Although contemporary clinical practice increasingly favors universal or self-etch adhesives, etch-and-rinse systems remain widely used in laboratory bond durability research because they allow controlled phosphoric acid demineralization followed by resin infiltration into an exposed collagen matrix. This characteristic makes them particularly suitable for evaluating how biomimetic pretreatment of demineralized dentin may influence resin infiltration and hybrid layer formation under defined conditions.

Despite the promising potential of biomimetic remineralization strategies, limited evidence is available regarding their influence on the bonding performance of etch-and-rinse adhesive systems over time. While self-assembling peptides and polymer-induced liquid precursor techniques have demonstrated effective remineralization of demineralized dentin, their impact on micro-tensile bond strength durability remains unclear. Therefore, the aim of the present study was to evaluate the effect of self-assembling peptide P11-4 and polymer-induced liquid precursor treatment on the micro-tensile bond strength of an etch-and-rinse adhesive to demineralized dentin at different storage periods.

The null hypothesis tested was that guided dentin remineralization would not affect the micro-tensile bond strength of an etch-and-rinse adhesive to demineralized dentin, regardless of the remineralization approach or storage time.

The micro-tensile bond strength results and failure mode distributions are illustrated in Figures 1–4.

## 2 Materials

The materials used in this study along with their commercial names, compositions, manufacturers, and lot numbers are listed in Table 1.

**Table 1:** Materials used.

Material	Commercial Name	Description	Composition	Manufacturer	Lot Number
Etchant	Meta Etchant	37% phosphoric acid gel for enamel and dentin etching	37% Phosphoric Acid Gel	Meta Biomed, Cheongju, S. Korea	MFG#: 309990
Bonding System	Adper Single Bond 2	Total-etch, light-cured dental adhesive	Ethanol-based adhesive with nanofillers	3M ESPE, St. Paul, MN, USA	REF: 51202
Resin Composite	Filtek Z250 XT	Nanohybrid universal restorative composite	Bis-GMA, UDMA, Bis-EMA resins with zirconia/silica fillers	3M ESPE, St. Paul, MN, USA	REF: 1470B1
Self-Assembling Peptide	Curodont Repair	Peptide-based agent for enamel remineralization	P11-4 self-assembling peptide	Credentis AG, Altendorf, Switzerland	NDC: 72247-0106
PILP Agent	L-Aspartic Acid Sodium Salt	Anionic polymer for stabilizing ACP in PILP process	L-Aspartic Acid Sodium Salt Monohydrate	Sigma-Aldrich, Buchs, Switzerland	Lot: WXBF4885V
De-mineralizing Solution	Carboxymethylcellulose (CMC)	Viscous agent for creating artificial lesions	Sodium Carboxymethylcellulose	Sigma-Aldrich, St. Louis, MO, USA	Lot: SLBQ1234V
Artificial Saliva Solution	N/A	Simulated saliva for specimen storage	Mixture of NaH₂PO₄, NaCl, KCl, CaCl₂·2H₂O, Na₂S·9H₂O, (NH₄)₂SO₄, citric acid, NaHCO₃, urea	Prepared in-lab	N/A

**Table 2:** The mean, standard deviation (SD) values and results of two-way ANOVA test for comparison.

Time	Control		Curodont		PASP		*P*-value	Effect size (*Partial eta squared*)
	Mean	SD	Mean	SD	Mean	SD		
1 day	14.66^B^	2.45	22.18^A^	3.71	15.43^B^	4.89	<0.001*	0.329
3 days	13.33^B^	3.24	21.26^A^	2.89	13.44^B^	2.07	<0.001*	0.371
14 days	3.81^A^	1.25	3.59^B^	1.4	5.12^B^	2.12	<0.001*	0.439

Different uppercase letters (A, B)indicate statistically significant differences between groups within the same row (*p* < 0.05).

^*^Indicates statistically significant difference (*p* ≤ 0.05).

**Table 3:** Descriptive statistics and results of fisher's exact test for comparison between failure modes in the three groups.

Time	Failure mode	Control		Curodont		PASP		*P*-value	Effect size (*Partial eta squared*)
		N	%	N	%	N	%		
1 day	Adhesive	1	16.7	0	0	2	33.3	**0** **.** **855**	**0** **.** **273**
	Cohesive Dentin	1	16.7	2	33.3	1	16.7		
	Mixed	4	66.7	4	66.7	3	50		
3 days	Adhesive	0	0	1	16.7	1	16.7	**0** **.** **735**	**0** **.** **307**
	Cohesive Dentin	0	0	1	16.7	0	0		
	Mixed	6	100	4	66.7	5	83.3		
14 days	Adhesive	0	0	5	83.3	6	100	**0** **.** 002*	**0** **.** **642**
	Cohesive Dentin	2	33.3	0	0	0	0		
	Mixed	4	66.7	1	16.7	0	0		

Bold values indicate statistically significant differences between groups.

## 3 Methods

This *in vitro* experimental study evaluated the effect of two biomimetic dentin pretreatment protocols on the micro-tensile bond strength (µTBS) of an etch-and-rinse adhesive to artificially demineralized dentin over different storage periods (9).

### 3.1 Experimental design

The study followed a 3 × 3 factorial design. The factors were:

1. **Dentin pretreatment (P)** with three levels:
  ◦ P1: No pretreatment (control)
  ◦ P2: Self-assembling peptide P11-4 (Curodont Repair™)
  ◦ P3: Polymer-induced liquid precursor (PILP) solution
2. **Storage time (T)** in artificial saliva with three levels:
  ◦ T0: 24 h
  ◦ T1: 3 days
  ◦ T2: 14 days

The primary outcome was micro-tensile bond strength (µTBS, MPa). Failure mode distribution was considered a secondary outcome. The experimental unit was the **tooth** (one beam specimen obtained from each tooth; *n* = 6 per treatment–time subgroup).

All experimental procedures, including dentin preparation, pretreatment application, adhesive restoration, specimen sectioning, and bond strength testing, were performed by a single calibrated operator to ensure procedural consistency. Due to the nature of the interventions, operator blinding during pretreatment application was not feasible. However, statistical analysis was conducted using coded group allocation to minimize potential bias.

### 3.2 Tooth selection and preparation

A total of **54 sound, caries-free, and periodontally unaffected human upper and lower first molars** were selected for this study. Teeth were extracted at Future University Dental Hospital. After extraction, each molar was cleaned to remove soft tissue remnants and calculus deposits and stored in distilled water at room temperature. All teeth were used within three months of extraction to maintain structural integrity.

The 54 teeth were randomly allocated into three main groups (*n* = 18 per group) according to dentin pretreatment protocol:

1. **Group 1 (Control, P1):** Demineralized dentin without pretreatment
2. **Group 2 (P2):** Demineralized dentin treated with Curodont Repair™ (P11-4)
3. **Group 3 (P3):** Demineralized dentin treated with PILP solution

Each main group was further subdivided according to storage time in artificial saliva at 37 °C (*n* = 6 per subgroup):

1. T0: Restored after 24 h
2. T1: Restored after 3 days
3. T2: Restored after 14 days

### 3.3 Dentin surface preparation

Each tooth was embedded in self-curing acrylic resin up to the cementoenamel junction, leaving the crown exposed. The occlusal enamel was removed using an automatic bench lathe machine under water cooling to obtain a flat mid-coronal dentin surface perpendicular to the long axis of the tooth. Surfaces were inspected to ensure uniform dentin exposure without enamel remnants.

### 3.4 Artificial dentin demineralization

A demineralizing gel was prepared using 6% sodium carboxymethylcellulose (CMC) dissolved in deionized water. Acetic acid was added and adjusted to a pH of approximately 4.5. Calcium chloride (CaCl_2_) and sodium dihydrogen phosphate (NaH_2_PO_4_) were incorporated to simulate physiological ionic conditions. Sodium benzoate was added as a preservative. Dentin surfaces were fully immersed in the 6% CMC acidic gel for 48 h to induce controlled demineralization. Specimens were then rinsed thoroughly with distilled water to remove residual gel (10).

### 3.5 Pretreatment protocols

Specimens were treated according to group allocation:

1. **P1 (Control):** No pretreatment was applied.
2. **P2 (Curodont Repair™, P11-4):** The self-assembling peptide P11-4 was applied using the manufacturer-supplied applicator and left undisturbed on the dentin surface for 5 min (7). Excess material was gently removed with paper tissue.
3. **P3 (PILP solution):** A polymer-induced liquid precursor solution was applied using a microbrush and left on the dentin surface for 1 h (11). Excess solution was removed with paper tissue.

The PILP solution was prepared at the Faculty of Pharmacy, Future University in Egypt. L-aspartic acid sodium salt (Sigma-Aldrich; MW ∼2,700 Da) was dissolved at 100 μg/mL in Tris buffer (pH 7.4). Calcium chloride dihydrate (CaCl_2_·2H_2_O) was added to a final concentration of 4.5 mM, followed by dropwise addition of potassium phosphate monobasic (KH_2_PO_4_) to achieve 2.1 mM. The solution was allowed to equilibrate for 30 min to form a stabilized amorphous calcium phosphate phase (12).

### 3.6 Storage protocol

After pretreatment, specimens were stored in artificial saliva at 37 °C for the designated periods (T0, T1, T2). Artificial saliva consisted of sodium dihydrogen phosphate monohydrate (780 mg/L), sodium chloride (500 mg/L), potassium chloride (500 mg/L), calcium chloride dihydrate (795 mg/L), sodium sulfide nonahydrate (5 mg/L), ammonium sulfate (300 mg/L), citric acid (5 mg/L), sodium bicarbonate (100 mg/L), and urea (1,000 mg/L). The pH was adjusted to 6.5 (13). Specimens were not sectioned prior to storage in order to maintain structural integrity of the bonded interface and to avoid premature exposure of dentin surfaces to dehydration or environmental changes. Sectioning after restoration ensured standardized bonding conditions across specimens and minimized potential alterations in beam geometry or moisture content that could influence micro-tensile bond strength measurements.

### 3.7 Adhesive and restorative procedures

The dentin surfaces were etched with 37% phosphoric acid (Meta Etchant, Meta Biomed) for 15 s, rinsed thoroughly, and gently air-dried. Adper Single Bond 2 adhesive (3M ESPE, USA) was applied according to the manufacturer's instructions, gently air-thinned, and light-cured for 10 s. Filtek Z250 XT nanohybrid resin composite (3M ESPE) was incrementally placed in five 1-mm layers (14, 15). Each increment was light-cured for 40 s using an LED curing unit (1,200 mW/cm^2^).

### 3.8 Micro-Tensile bond strength testing

After restoration, specimens were sectioned longitudinally using a diamond disc (0.3 mm thickness) under water cooling. Sectioning was performed in bucco-lingual and mesio-distal directions to obtain rectangular beam specimens with approximate cross-sectional dimensions of 1 mm × 1 mm, verified using a digital caliper. One beam specimen was obtained from each tooth, resulting in a total of **54 beam specimens (*n*** **=** **6 per subgroup)**.

Each beam was fixed to a custom testing jig using cyanoacrylate adhesive and mounted in a universal testing machine equipped with a 500 N load cell. Tensile load was applied at a cross-head speed of 0.5 mm/min until failure occurred. Bond strength values were calculated in megapascals (MPa) by dividing the maximum load at failure by the cross-sectional area.

### 3.9 Failure mode analysis

Fractured specimens were examined under a stereomicroscope at ×40 magnification. Failure modes were classified as:

1. Adhesive failure
2. Cohesive failure in dentin
3. Cohesive failure in resin composite
4. Mixed failure

### 3.10 Statistical analysis

Statistical analysis was performed using IBM SPSS Statistics for Windows. Normality of data distribution was assessed using Kolmogorov–Smirnov and Shapiro–Wilk tests. Micro-tensile bond strength values showed normal distribution and were expressed as mean ± standard deviation (SD). In accordance with recommendations for micro-tensile bond strength studies and guidance from the Academy of Dental Materials (Armstrong et al., Dental Materials, 2016), the tooth was considered the experimental unit to avoid clustering effects (16). One beam specimen was obtained from each tooth. Two-way ANOVA was used to evaluate the effects of dentin pretreatment, storage time, and their interaction on µTBS values. Bonferroni *post-hoc* tests were applied for pairwise comparisons when significant differences were detected (*α* = 0.05). Failure mode distributions were analyzed using Fisher's Exact test. The significance level was set at *P* ≤ 0.05.

## 4 Results

The descriptive statistics and failure mode distributions are presented in Tables 2 and 3.

### 4.1 Micro-tensile bond strength (µTBS)

#### 4.1.1 Two-way ANOVA

Two-way ANOVA revealed that dentin pretreatment (P), storage time (T), and their interaction (*P* × T) had statistically significant effects on micro-tensile bond strength (*p* < 0.001). Because a significant interaction was detected, the effects of dentin pretreatment were analyzed within each storage time, and the effect of storage time was analyzed within each pretreatment group.

#### 4.1.2 Effect of pretreatment within each storage time

At **24 h (T0)**, there was a statistically significant difference among groups (*p* < 0.001). The P11-4 group demonstrated the highest mean µTBS (22.18 ± 3.71 MPa), which was significantly higher than both the control group (14.66 ± 2.45 MPa) and the PILP group (15.43 ± 4.89 MPa). No statistically significant difference was observed between the control and PILP groups.

At **3 days (T1)**, a statistically significant difference was also observed (*p* < 0.001). The P11-4 group again showed the highest mean µTBS (21.26 ± 2.89 MPa), significantly greater than both the control group (13.33 ± 3.24 MPa) and the PILP group (13.44 ± 2.07 MPa). No significant difference was found between control and PILP.

At **14 days (T2)**, statistically significant differences were present among groups (*p* < 0.001). The control group exhibited the highest mean µTBS (3.81 ± 1.25 MPa), while both P11-4 (3.59 ± 1.40 MPa) and PILP (5.12 ± 2.12 MPa) groups demonstrated significantly reduced bond strength compared with earlier time points. No statistically significant difference was detected between P11-4 and PILP at 14 days.

#### 4.1.3 Effect of storage time within each pretreatment group

Within the **control group**, µTBS values significantly decreased over time (*p* < 0.001), with the lowest values recorded at 14 days. Within the **P11-4 group**, µTBS values at 24 h and 3 days were not significantly different from each other; however, both were significantly higher than values recorded at 14 days (*p* < 0.001). Similarly, within the **PILP group**, bond strength values at 24 h and 3 days were comparable, but a significant reduction was observed at 14 days (*p* < 0.001). These findings indicate that the influence of biomimetic pretreatment on bond strength was time-dependent.

### 4.2 Failure mode analysis

At **24 h (T0)** and **3 days (T1)**, no statistically significant differences were detected in failure mode distribution among groups (*p* > 0.05). Mixed failures predominated across all groups at these time intervals. At **14 days (T2)**, a statistically significant difference in failure mode distribution was observed (*p* = 0.002). The PILP group exhibited predominantly adhesive failures (100%), followed by the P11-4 group (83.3% adhesive failures). In contrast, the control group demonstrated no adhesive failures at 14 days, with a higher prevalence of mixed failures (66.7%) and cohesive failures in dentin (33.3%).

## 5 Discussion

The present study evaluated the influence of two biomimetic dentin pretreatment strategies—self-assembling peptide P11-4 and the polymer-induced liquid precursor (PILP) approach—on the micro-tensile bond strength of an etch-and-rinse adhesive to artificially demineralized dentin over different storage periods. A significant interaction between pretreatment and storage time was observed, indicating that the effect of biomimetic treatment was time-dependent.

At 24 h and 3 days, pretreatment with P11-4 resulted in significantly higher bond strength compared with both the control and PILP groups. This finding suggests that P11-4 modified the dentin substrate in a manner that initially favored adhesive bonding. However, it is essential to distinguish between biomimetic remineralization as a biological objective and adhesive bonding as a micromechanical process. Etch-and-rinse systems depend on controlled phosphoric acid demineralization followed by resin infiltration into an exposed collagen network. Therefore, any pretreatment capable of altering collagen structure, surface energy, mineral distribution, or moisture dynamics may influence bond strength independently of confirmed intrafibrillar remineralization.

The higher early µTBS values observed with P11-4 cannot be definitively attributed to true intrafibrillar mineral deposition, as no ultrastructural or compositional analyses (e.g., SEM/EDS, TEM, FTIR, micro-CT, or nanoindentation) were performed in the present study. The observed enhancement may alternatively be explained by temporary surface mineral precipitation, modification of substrate wettability, increased collagen stiffness, or potential interaction between peptide residues and adhesive monomers. Previous studies have demonstrated that P11-4 can function as a nucleation scaffold for hydroxyapatite formation (7, 17, 18); however, the direct relationship between peptide-mediated mineral deposition and immediate adhesive bond strength remains incompletely elucidated.

The PILP-treated group demonstrated bond strength values comparable to the control group at 24 h and 3 days. The PILP process has been reported to promote intrafibrillar mineral deposition through stabilization of amorphous calcium phosphate nanodroplets (12, 19, 20). Nevertheless, remineralization of collagen does not necessarily translate into improved hybrid layer formation. If mineral deposition occurs superficially or partially occludes interfibrillar spaces, resin infiltration may be hindered. This potential mechanistic mismatch between remineralization and bonding may explain the absence of superior performance in the PILP group at early time points.

The application protocols used in the present study were based on previously published *in vitro* remineralization methodologies (11, 12), allowing sufficient interaction between the remineralizing agents and demineralized collagen. However, the 5-minute application time for P11-4 and particularly the 1-hour application time for the PILP solution may limit immediate clinical feasibility. Extended exposure of demineralized dentin to aqueous remineralizing environments prior to adhesive application could increase dentin moisture content, alter collagen mechanical properties, or modify interfibrillar spacing. Such changes may influence subsequent resin infiltration, especially in etch-and-rinse systems that rely on controlled collagen exposure. Accordingly, while these protocols are appropriate for mechanistic laboratory evaluation, their direct translation to routine clinical practice should be approached with caution.

At 14 days, all groups exhibited a significant reduction in bond strength. The decrease was particularly evident in the biomimetic groups, and failure mode analysis revealed a predominance of adhesive failures in P11-4 and PILP specimens. In contrast, the control group demonstrated mixed and cohesive-in-dentin failures at the same interval. This shift in failure pattern supports the quantitative findings and suggests progressive weakening of the adhesive interface over time in the pretreated groups.

The reduction in bond strength after prolonged storage may be attributed to hydrolytic degradation of the hybrid layer, water sorption, and collagen matrix destabilization, as previously described by Hashimoto et al. (21) and Carrilho et al. (22). In biomimetically treated dentin, it is also plausible that loosely integrated or superficially deposited mineral phases were unstable under aqueous conditions. If mineral phases were not fully integrated within collagen fibrils, their dissolution or degradation during storage could have compromised interfacial integrity.

Importantly, the present findings challenge the assumption that biomimetic remineralization inherently enhances adhesive durability. Although biomimetic strategies aim to restore mineral content within demineralized collagen, adhesive bonding requires open interfibrillar spaces to allow adequate resin infiltration. Partial remineralization prior to bonding could theoretically reduce substrate permeability or alter porosity, potentially hindering resin diffusion in etch-and-rinse systems. Thus, remineralization and adhesive bonding should not be regarded as mechanistically equivalent objectives.

The adhesive used in this study (Adper Single Bond 2) is an ethanol-based etch-and-rinse system containing hydrophilic and hydrophobic monomers. Alterations in dentin moisture or surface mineral distribution induced by biomimetic pretreatment may have influenced solvent evaporation dynamics and resin infiltration behavior. Such interactions may partially explain the time-dependent changes observed in bond strength.

Previous investigations have reported improved long-term bonding with selective remineralizing agents (17, 18), whereas others have demonstrated variable or neutral effects depending on formulation, lesion model, and aging protocol (23). Differences in substrate characteristics, application duration, storage conditions, and adhesive category likely contribute to heterogeneity among studies. Previous studies have reported variable effects of biomimetic remineralization strategies on adhesive bond durability depending on the application protocol, substrate characteristics, and adhesive system used (24–28). The present study employed a single adhesive system and a single application protocol; therefore, extrapolation to universal or self-etch adhesives should be made cautiously.

From a clinical perspective, the early increase in bond strength observed with P11-4 may offer potential benefits in minimally invasive restorative approaches involving demineralized dentin. However, the marked reduction in bond strength after 14 days underscores the importance of evaluating long-term stability before incorporating additional pretreatment steps into adhesive protocols. Moreover, the extended application time required for the PILP protocol may limit its practical applicability. The cost–benefit ratio and procedural complexity must be considered prior to clinical translation.

Several limitations must be acknowledged. First, this was an *in vitro* investigation, and artificial demineralization combined with static storage in artificial saliva does not replicate the dynamic oral environment, including thermal cycling, enzymatic activity, pH fluctuations, and masticatory stresses. Second, only bond strength and failure mode were assessed. Failure mode analysis performed under ×40 stereomicroscopy provides limited insight into ultrastructural interfacial integrity. Third, no direct structural confirmation of remineralization was performed, and mechanistic interpretations therefore remain speculative. Additionally, the chemically induced demineralization model may not fully reproduce the heterogeneity and tubular characteristics of natural caries-affected dentin. Finally, the aging period was limited to 14 days, representing short-term evaluation.

Future investigations incorporating longer aging intervals, thermomechanical cycling, enzymatic challenges, and high-resolution ultrastructural analyses are necessary to clarify whether controlled biomimetic strategies can enhance adhesive durability without compromising resin infiltration. Evaluation of these approaches in combination with bioactive or collagen-stabilizing adhesive systems may also provide further insight.

Within the limitations of this study, guided dentin pretreatment influenced micro-tensile bond strength in a time-dependent manner. Early improvements observed with P11-4 did not translate into superior performance after prolonged storage. These findings suggest that biomimetic remineralization does not inherently guarantee enhanced adhesive durability and that careful consideration of the interaction between substrate modification and adhesive mechanism is required.

## 6 Conclusion

Within the limitations of this *in vitro* study, dentin pretreatment with biomimetic strategies significantly influenced the micro-tensile bond strength of an etch-and-rinse adhesive to artificially demineralized dentin in a time-dependent manner. The self-assembling peptide P11-4 demonstrated significantly higher bond strength at early storage periods (24 h and 3 days) compared with the PILP approach and control. However, after 14 days of storage, all groups exhibited a marked reduction in bond strength, and no sustained superiority of biomimetic pretreatment was observed. These findings indicate that while biomimetic dentin pretreatment may transiently modify substrate characteristics and enhance early bond strength, it does not inherently ensure improved bond durability under aqueous aging conditions. Further studies incorporating long-term aging protocols and ultrastructural evaluation are required to clarify the relationship between biomimetic remineralization and adhesive performance.

## Data Availability

The datasets presented in this article are not readily available because no restrictions. Requests to access the datasets should be directed to 20206527@fue.edu.eg.
